# The effect of nurse practitioner (NP-led) care on health-related quality of life in people with multiple sclerosis – a randomized trial

**DOI:** 10.1186/s12883-022-02809-9

**Published:** 2022-07-25

**Authors:** Penelope Smyth, Kaitlyn E. Watson, Yazid N. Al Hamarneh, Ross T. Tsuyuki

**Affiliations:** 1 Division of Neurology, Department of Medicine, University of Alberta, Edmonton, Canada; 2 EPICORE Centre, Department of Medicine, University of Alberta, Edmonton, Canada

**Keywords:** Multiple sclerosis, Nurse practitioners, Hospital anxiety and depression scale, Depression, Anxiety, Quality of life, General neurologists

## Abstract

**Background:**

Care for People with Multiple Sclerosis (PwMS) is increasingly complex, requiring innovations in care. Canada has high rates of MS; it is challenging for general neurologists to optimally care for PwMS with busy office practices. The aim of this study was to evaluate the effects of add-on Nurse Practitioner (NP)-led care for PwMS on depression and anxiety (Hospital Anxiety and Depression Scale, HADS), compared to usual care (community neurologist, family physician).

**Methods:**

PwMS followed by community neurologists were randomized to add-on NP-led or Usual care for 6 months. Primary outcome was the change in HADS at 3 months. Secondary outcomes were HADS (6 months), EQ5D, MSIF, CAREQOL-MS, at 3 and 6 months, and Consultant Satisfaction Survey (6 months).

**Results:**

We recruited 248 participants; 228 completed the trial (NP-led care arm *n* = 120, Usual care arm *n* = 108). There were no significant baseline differences between groups. Study subjects were highly educated (71.05%), working full-time (41.23%), living independently (68.86%), with mean age of 47.32 (11.09), mean EDSS 2.53 (SD 2.06), mean duration since MS diagnosis 12.18 years (SD 8.82) and 85% had relapsing remitting MS. Mean change in HADS depression (3 months) was: -0.41 (SD 2.81) NP-led care group vs 1.11 (2.98) Usual care group *p* = 0.001, sustained at 6 months; for anxiety, − 0.32 (2.73) NP-led care group vs 0.42 (2.82) Usual care group, *p* = 0.059. Other secondary outcomes were not significantly different. There was no difference in satisfaction of care in the NP-led care arm (63.83 (5.63)) vs Usual care (62.82 (5.45)), *p* = 0.194).

**Conclusion:**

Add-on NP-led care improved depression compared to usual neurologist care and 3 and 6 months in PwMS, and there was no difference in satisfaction with care. Further research is needed to explore how NPs could enrich care provided for PwMS in healthcare settings.

**Trial registration:**

Retrospectively registered on clinicaltrials.gov (ClinicalTrials.gov Identifier: NCT04388592, 14/05/2020).

**Supplementary Information:**

The online version contains supplementary material available at 10.1186/s12883-022-02809-9.

## Introduction

Multiple Sclerosis (MS) is the leading cause of non-traumatic disability in young adults [1]. It is most commonly diagnosed in early to mid-adulthood, causing visible symptoms such as vision difficulties, gait troubles, weakness, coordination difficulties, and/or invisible symptoms such as fatigue, cognitive decline, depression, anxiety, bladder and bowel issues, sexual impairment, and pain [2, 3]. MS most commonly follows a relapsing-remitting course, ultimately transitioning into secondary progression MS as progression of disability advances, or following a progressive course from onset, identified as primary progressive MS. [4]. There is no cure for MS, and disability usually progresses over a person’s lifetime [2–4]. MS prevalence is increasing over time, with Canada having one of the highest incidence rates of MS in the world [1, 3]. Within Canada, Alberta has the highest prevalence of MS (340 per 100,000 population) [2], with 14,000 Albertans living with MS in 2013 [2].

Being diagnosed and living with MS is challenging, stressful, and unique for every person with MS and their caregivers; this requires individualized and ongoing support from healthcare providers and multi-disciplinary teams [5–7]. Timely access to specialized care is essential in diagnosing MS, monitoring disease, and managing people with MS (PwMS), as identified by an international expert group consensus around international quality standards of care for PwMS in 2019 [8]. Delays and difficulties in making appointments with neurologists have been identified as key barriers interfering with optimized care delivery to PwMS [9]. Indeed, in Canada, a recent online survey of Canadians living with MS (*n* = 324 responders) reported that 70% experienced challenges in obtaining appointments with their neurologists [10]. Even when meeting with healthcare providers, multiple studies reported that PwMS do not receive enough education or support from their health-care providers in order to meet their needs [11–24]. This may be evolving in Canada, as suggested by Petrin et al. in 2021, where survey respondents expressed high levels of satisfaction with their healthcare providers around respectful communication and in shared decision making [25].

PwMS require decades of specialized neurologic care, with average life expectancy of one large Canadian cohort estimated to be approximately 6 years less than the general population [26]. A cross-sectional questionnaire study of 1205 PwMS identified unmet needs for PwMS as changing and evolving as their disability progresses [18]. Therefore, MS experts have emphasized that healthcare providers and services need to adapt to the different stages and health needs of PwMS – from early diagnosis to elderly patients [5, 8, 9, 18, 19]. This might be difficult to achieve in the current model of care where community general neurologists have very busy office practices [7, 27]. Specialized care within multi-disciplinary “MS Units”, including a specialized MS nurse as a key player within the team, has been suggested to fill these types of gaps [7].

A systematic review published in 2015 compared NP-led care to usual care for people with chronic diseases. Limited evidence inferred that NP involvement improved patient outcomes [27]. Some NP-led care trials utilized the NP instead of a physician specialist as a specialized, independent healthcare provider, backed up by a physician specialist [28–30]. In other trials, the NP worked as an add-on provider to the multi-disciplinary ambulatory care setting [31–34]. One randomized trial involving 122 participants with Parkinson’s disease compared outcomes of care delivered by a multi-disciplinary team (including a specialized nurse) to general neurologists over an eight-month period. Patients receiving care from the multi-disciplinary team reported improved scores on quality of life (QoL) and Parkinson’s scale outcomes [35].

Private-practice, community general neurologists and family physicians provide care for approximately 2000 PwMS outside of a multidisciplinary MS specialized clinic in northern Alberta, Canada. Usual care involves biannual to annual office visits, with variable referral to rehabilitation services and therapies, as determined by clinical judgement. Pressures on general neurologists and family physicians in busy office practices, combined with the treatment gaps and unmet needs of PwMS, underline the need for alternative ways to provide care. Adding a specialized MS NP as an additional healthcare provider to generalist neurology and primary care could potentially improve care for PwMS. Alternate specialized healthcare providers such as nurse practitioners (NPs) could be helpful in delivering care to PwMS in the Canadian healthcare setting. Further information about the NP model is available in the study protocol paper [36].

The primary objective was to evaluate the impact of an add-on NP-led care for PwMS and their caregivers on depression and anxiety levels.

## Methods

### Study design

We conducted a prospective parallel randomized controlled trial (RCT) with patients as the unit of randomization, with allocation ratio 1:1. The rationale and protocol for this RCT have been reported previously [36].

### Setting

We included 7 community neurologist (CN) practices across Edmonton, Alberta, Canada and the tertiary MS Clinic at the University of Alberta Hospital.

### Ethics approval

The study was performed in accordance with the Declaration of Helsinki, and was approved by the Health Research Ethics Boards of the University of Alberta (approval number Pro00069595). This trial was registered retrospectively on clinicaltrials.gov (ClinicalTrials.gov Identifier: NCT04388592, 14/05/2020).

### New clinical tools and procedures

The introduction of the NP into community general neurologist care for PwMS is novel. However, NP care for PwMS is standard at tertiary care centres and MS Clinics in optimizing care for PwMS.

### Consent to participate

Informed consent was obtained from all participants to participate in the study, prior to being randomized to participate within the study, and before completing baseline measures.

### Population

Patients were included in the study if they were adults (≥ 18 years) who had a diagnosis of MS as per the McDonald 2010 criteria [37], were followed by a private-practice general neurologist and/or family doctor, willing to give consent, able to complete questionnaires and to attend outpatient visits with NP, English-speaking, and able to use a computer.

We excluded patients if they were unable to provide consent, unable or unwilling to attend appointments, referred to, or followed by neurologists within the tertiary setting, or had other central nervous system inflammation disorders.

### Recruitment

Participants were recruited through advertisements and computer tablets were placed in the waiting rooms of the seven CN practices. Interested patients completed the EQ5D [38] on a tablet and were encouraged to self-register for the study and discuss their results with their CN. We also accepted direct referrals from family physicians to the NP. Details about the recruitment process are available elsewhere [36]. All participants provided informed consent to participate in the trial.

### Randomization and blinding

After being enrolled in the study, patients were randomized in a 1:1 ratio to either the intervention or control groups [36]. Due to the nature of the intervention, blinding of providers or patients was not possible. The EPICORE Centre randomly randomized and allocated consented participants using a centralized secure website. Further details can be found in the study protocol publication [36].

### Intervention

**Fig. 1:**
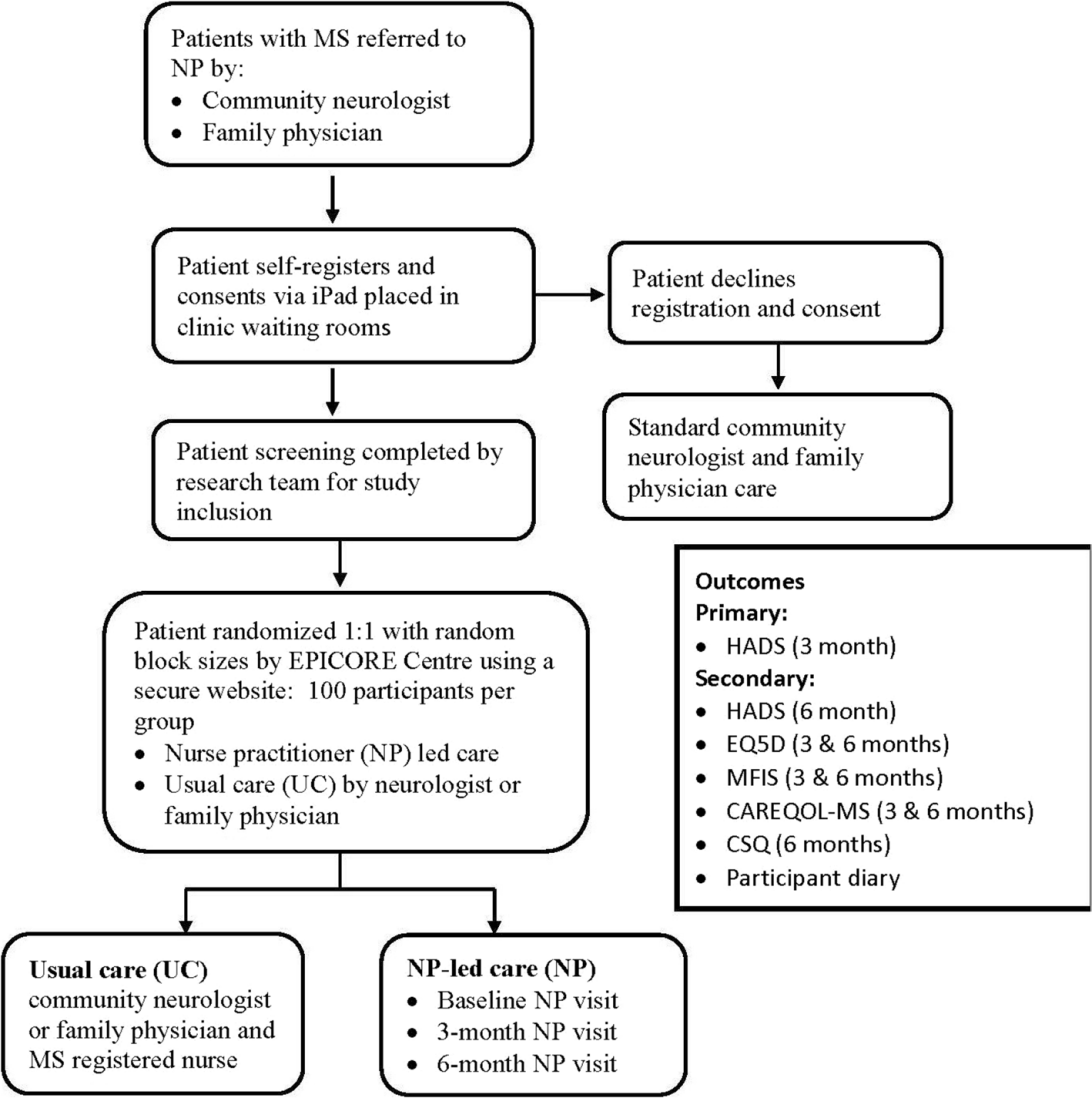
Outline of care and tests provided to PwMS involved in this study

Patients in the intervention group received a comprehensive NP consultation. This included 1) patient history, 2) physical examination, 3) individualized symptomatic strategies (e.g., lifestyle strategies, mobility issues, fatigue, spasticity, bladder and bowel concerns, depression or anxiety, and medications) [39, 40], 4) exploration of the MS patient’s local community, 5) discussion of resources to optimize mood and QoL, and 6) regular follow up visits at 3, 6 months either in-person, via telehealth, or via phone call (Fig. 1). The NP carrying out the study intervention was mentored and trained by the experiences tertiary MS clinic NP, who has over 6 years of experience in MS, and the MS clinic subspecialist neurologists for a 3 month duration before the study was initiated, and 1 month after the study NP had worked in the community setting. In addition, the MS clinic NP and MS clinic subspecialist neurologists continued to be a resource for the study NP to contact with questions throughout the study duration. Please see the study protocol publication for further details [36].

### Control

Patients randomized to the control group received usual care from CN practices, which included limited access to MS registered nurses (part of the tertiary MS clinic setting but going to community neurologists’ offices as outreach). This care was delivered according to standard practices. Some general neurologists participating in our study, did have the option of having registered nurses aid them on a designated day once per week. These registered nurses had limited scope of practice in the community neurologists’ offices of rooming patients with MS, updating medication lists, and helping the neurologists with disease-modifying therapy initiation and renewals. Due to the limited scope of support, the registered nurses did not have the resources or time to support the community neurologists in targeted symptom management strategies and lifestyle strategies such as bladder and bowel management, relapse management, delivery of comprehensive education to PwMS. Follow-up visits were conducted according to the individual standard care practices (Fig. 1). Patients in the control group were offered the NP intervention after 6 months.

### Outcomes

The primary outcome was the difference in change in HADS-D and HADS-A scores between intervention (NP-led care) and control (Usual care) groups at 3 months [38, 41, 42]. Secondary outcomes included difference in change in a) HADS-D and HADS-A scores at 6 months, b) EQ5D at 3 and 6 months [38], and c) Modified Fatigue Impact Scale (MFIS) score at 3 and 6 months [43, 44], and patient satisfaction with care as measured by the validated Consultation Satisfaction Questionnaire (CSQ) [45–48].

### Sample size calculation

Using the information from Honarmand and Feinstein [41], [Baseline scores and standard deviation (SD)] and the following assumptions 80% power and a two-sided alpha of 0.025, a total sample size of 200 (100 in each group) was required to detect 1.5 difference [41] between the intervention and the control groups. We calculated the same size for both HADS-A and HADS-D and used the sample size for HADS-A, as it required a larger sample size and to ensure there was sufficient power for both HADS-A and HADS-D. This sample size was inflated to 220 to account for possible dropouts, losses to follow-up and withdrawals of consent.

### Statistical analysis

Data analysis was performed using R 3.4.0 computer software (Vienna, Austria; https://www.R-project.org/) and SAS 9.4 software (SAS Institute Inc. Cary, NC, USA). Patient demographic and clinical characteristics were analyzed using descriptive statistics. Categorical variables were reported using frequency and percentage and continuous variables were reported using mean (SD) or median [Interquartile range (IQR)] as appropriate. The primary outcome of difference in change of HADS-D and HADS-A from baseline to 3 months was analyzed using independent T-test. An independent T-test was also used to assess the difference in change between HADS-D and HADS-A between baseline and 6 months as well as the other questionnaires - EQ5D and MFIS. The CSQ Likert scales were analyzed as continuous variables, with the overall satisfaction score being calculated as a sum of the scales of each question and analyzed using an independent t-test to determine a difference between the intervention and control groups.

Data were analyzed according to the intention-to-treat principles. Trial and Data Management was completed by EPICORE Centre (www.epicore.ualberta.ca).

## Results

**Fig. 2:**
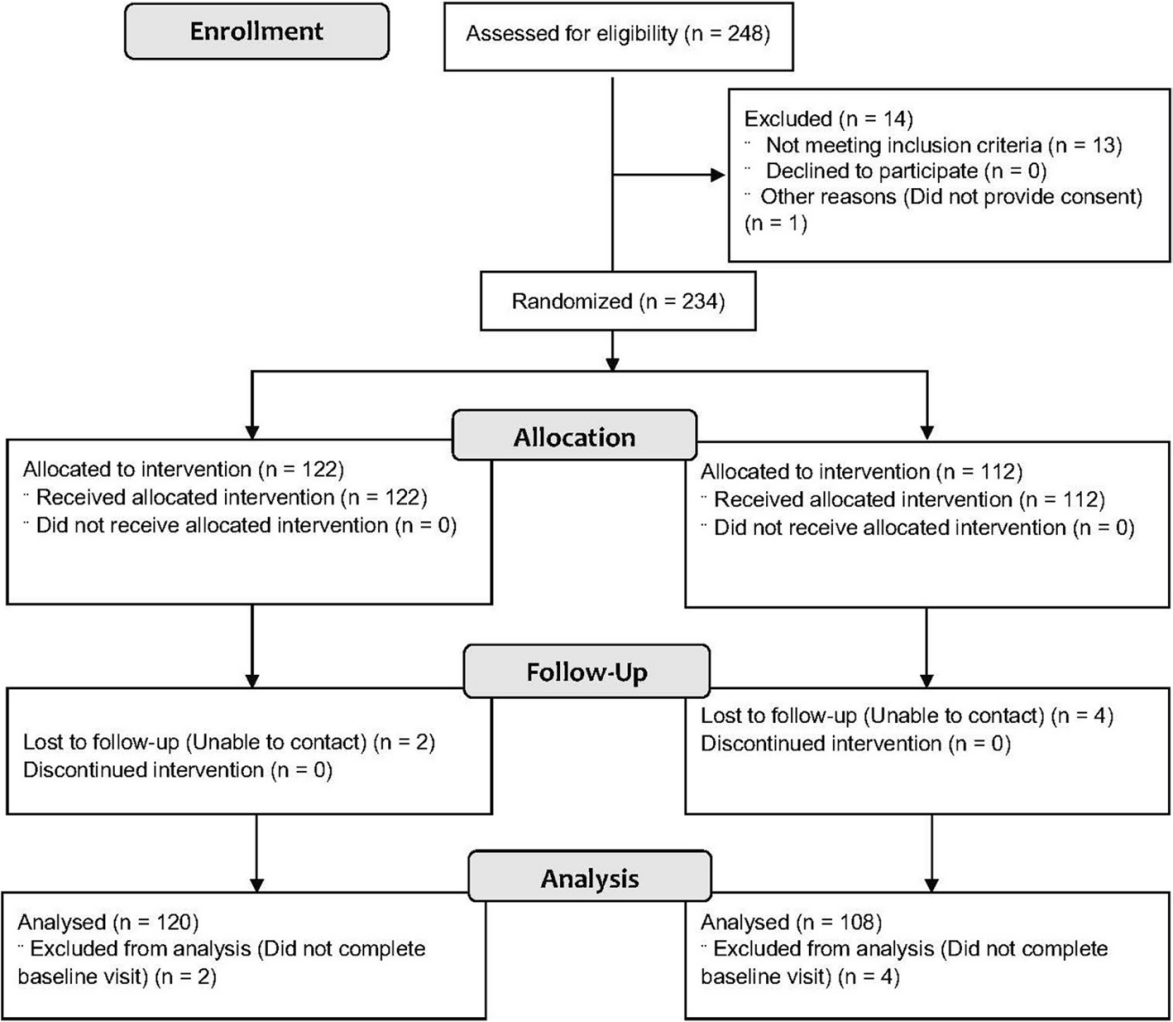
Trial flow using the Consort flow diagram

A total of 248 subjects were screened, of those, 234 were eligible to take part. All eligible patients provided informed consent and were enrolled in the study. Of those, 8 patients did not attend their baseline visit, and 226 patients completed the study and were included in the analysis (Fig. 2). Of note, although the study was open to PwMS followed primarily by either a community general neurologist, or a family physician, all participants in the study were followed by a general neurologist. Recruitment began on July 28, 2017 and was completed on March 3, 2019. The last participant in the study completed their surveys on Sept 25, 2019.

The 2 treatment groups were well balanced in baseline demographic and clinical parameters (Table 1). The mean age was 47.47 ± 11.02 years, 82.89% were female and 92.54% were white. More than two thirds of the participants were highly educated (71.05%) and living independently (68.86%), and less than half were working full time (41.23%).

**Table 1:** Baseline demographics and clinical characteristics

Baseline Measurement	Intervention (NP-led care) ***N*** = 118 (%)	Control (Usual care) ***N*** = 108 (%)	Total ***N*** = 228 (%)
**Demographics**			
Age, mean (SD)	46.89 (10.38)	47.80 (11.86)	47.47 (11.02)
Female sex, n (%)	97 (80.83)	92 (85.19)	189 (82.89)
Ethnicity, white n (%)	107 (89.17)	104 (96.3)	211 (92.54)
Married/Common-law n (%)	89 (74.17)	76 (70.37)	165 (72.37)
Education, community college or above n (%)	87 (72.5)	75 (69.44)	162 (71.05)
Full-time employment n (%)	50 (41.67)	44 (40.74)	94 (41.23)
Living at home (independently) n (%)	86 (71.67)	71 (65.74)	157 (68.86)
**Clinical features of MS**			
Type of MS, n (%)			
**-** relapsing-remitting MS (RRMS)	103 (85.83)	91 (84.26)	194 (85.09)
**-** primary-progressive MS (PPMS)	6 (5.00)	6 (5.56)	12 (5.26)
**-** Secondary-progressive MS (SPMS)	11 (9.17)	11 (10.19)	22 (9.65)
Duration of MS, mean (SD)	11.69 (8.42)	12.71 (9.24)	12.26 (8.81)
Disability scores, mean (SD)			
**-** Expanded Disability Status Scale (EDSS)	2.52 (2.07)	2.55 (2.07)	2.54 (2.07)
**-** Patient Determined Disease Steps (PDSS)	2.53 (2.01)	2.57 (2.05)	2.56 (2.03)
**-** Symbol Digit Modalities Test (SDMT) score	54.42 (11.68)	53.53 (13.04)	54.02 (12.36)

Values are n (%) or mean (SD)

### Primary outcome

**Fig. 3:**
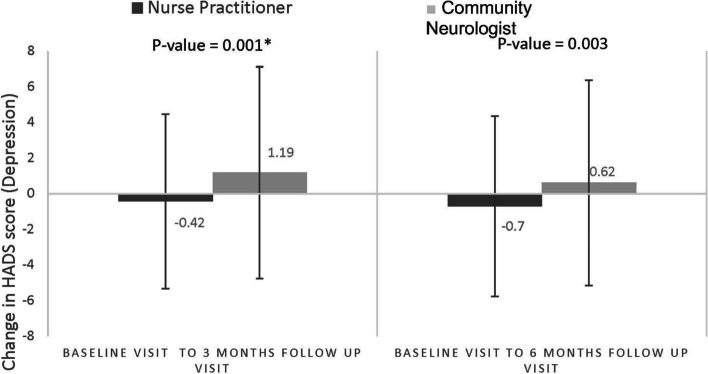
Difference in change of HADS-D over 3 and 6 months. *P-value was calculated by Wilcoxon signed-rank test

**Fig. 4:**
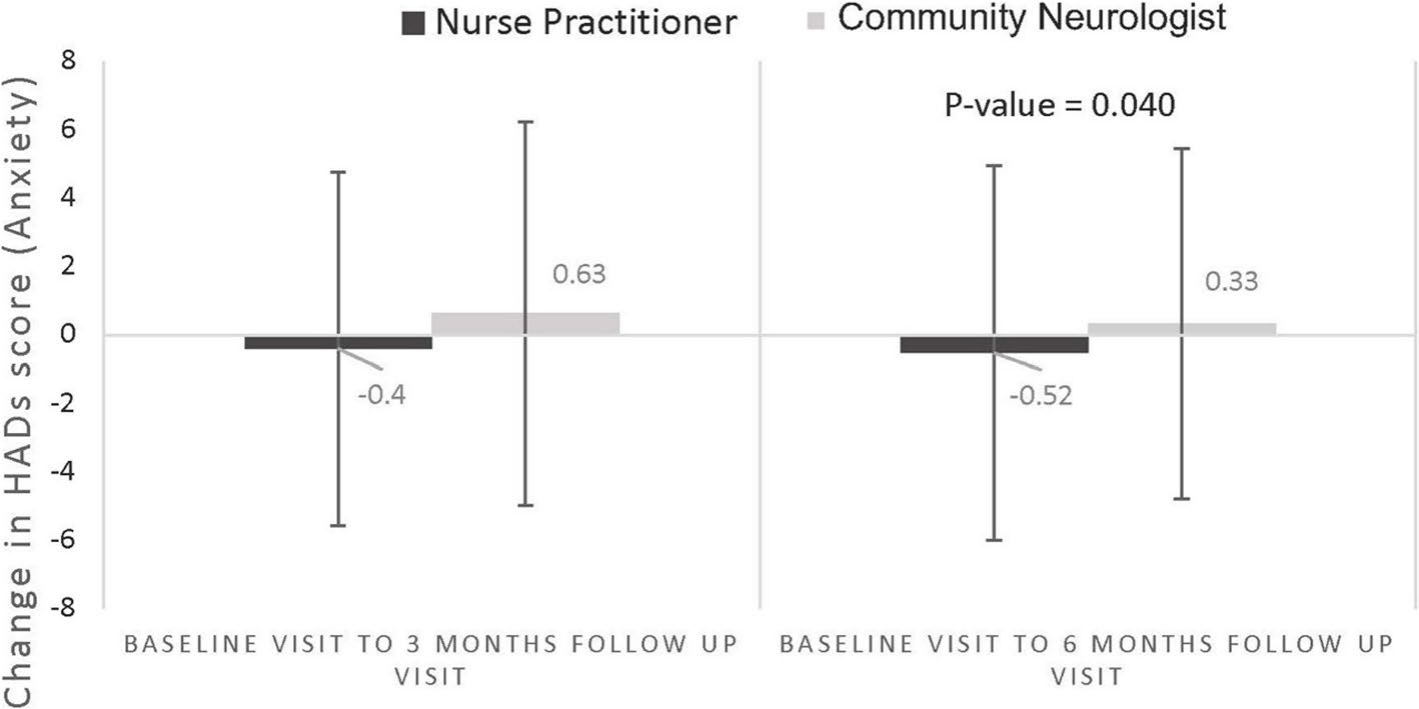
Difference in change of HADS-A over 3 and 6 months. *P-value was calculated by Wilcoxon signed-rank test

The mean difference in the change in HADS-D at 3 months was: -0.41 (SD 2.81) for the NP-led group vs 1.11 (2.98) Usual care group, *p* = 0.001 (Fig. 3); and for HADS-A, − 0.32 (2.73) NP-led group vs 0.42 (2.82) Usual care group, *p* = 0.059 (Fig. 4).

### Secondary outcomes

At 6 months, the mean change in HADS-D was − 0.81 (SD 3.18) for the NP-led group vs 0.57 (SD 3.11) Usual care group, *p* = 0.003 (Fig. 4); and for HADS-A -0.46 (3.18) NP-led group vs 0.36 (2.55) Usual care group, *p* = 0.04 (Fig. 4).

The difference in mean change for MFIS and EQ5D were not statistically significant (Table 2). The mean change for MFIS at 3 months was − 0.31 (9.77) for the NP group vs 0.97 (10.6) for UC group, *p* = 0.54. The mean change for the EQ5D score at 3 months was 1.58 (14.5) for the NP group vs − 0.02 (14.99) for UC group, *p* = 0.54. The mean change at 6 months for the MFIS and EQ5D showed a similar trend but was also not statistically significant.

**Table 2:**
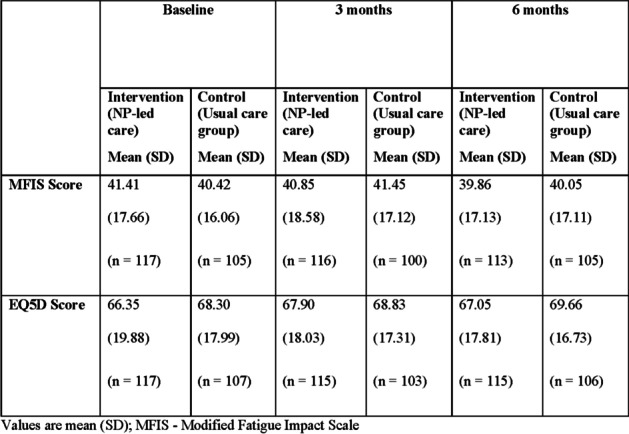
Changes in patient-reported outcome measures between NP-led care and Usual care at each time point – baseline, 3 months, and 6 months

There was no difference in satisfaction with care between the NP and Usual care groups. The mean overall satisfaction score for the NP-led group was 63.83 (5.63) and for the Usual care group was 62.82 (5.45), with a *p* value of 0.194. No statistical differences were noted on any of the sub-scales between the two groups (Additional file 1: Appendix A).

## Discussion

To our knowledge, this is the first study to examine the impact of an “add-on” specialized MS NP-led care to PwMS in comparison to usual care through community neurologist and family physicians’ practices. We found that NP-led care was associated with improved depression levels for PwMS at 3 months, sustained at 6 months, compared to usual physician care. At 6 months, an improvement in anxiety was observed. This study suggests that NPs could address many of the unmet needs identified by PwMS [22, 23]: improved education, coping strategies, more timely and effective interventions, urgent relapse management, in addition to helping PwMS optimize their functioning.

We looked at depression and anxiety levels for PwMS as primary and secondary outcome measures over the short-term intervention of 3 and 6 months. We felt depression and anxiety levels might be the most responsive factors over the short-term in relation to participants’ quality of life (QoL). Depression has been reported to influence PwMS’ QoL in multiple studies [49–58]. Overall, PwMS have reported a lower QoL compared to the general population [49, 53, 54, 56]. Initiating interventions to depression and anxiety fit within the MS-specialized NP scope of practice [6, 59–62]; depression and possibly anxiety in PwMS can respond to pharmacologic, and non-pharmacologic strategies [63–67]. Our study showed that PwMS reported statistically improved depression scores at 3 months, sustained at 6 months, when an NP was added. Depression is common in PwMS with an estimated prevalence between 14 to 54%, and a pooled mean prevalence of 30.5% (95%CI 26.3–35.1%) of depression, and 22.1% (95% CI 15.2–31.9%) in one systematic review and meta-analysis [68]. There are mixed findings amongst researchers as to whether there is increased depression with increased disability [50, 69], or not [68, 70–72], but it is common even at the time of diagnosis [72]. Furthermore, both anxiety and depression have been shown to be under-recognized and undertreated in PwMS in Canada [58, 73]. Despite statistically significant improvements in depression and anxiety scores in our study, further research on the minimal clinically important difference (MCID) score for PwMS is needed before conclusions can be drawn about the clinical significance of these findings.

It has been suggested that NPs might be easier to access and less costly to see than physician health providers for PwMS [6, 74–76]. The use of specialized MS NPs in general neurology outpatient settings could potentially address some of these unmet needs that various researchers have identified including healthcare access [10, 11, 22, 77–79], education, counseling and support [13, 79], and support to informal caregivers of PwMS [13, 79–81]. The need for more specialized MS nursing care has been identified as integral to improving patient care delivery for PwMS [6, 82].

There was no difference in level of satisfaction by PwMS in being cared for by a specialized NP compared to usual care received from community neurologists. This is in line with what has reported in the literature where the MS nurse was identified as the preferred medical contact [24, 83, 84]. An audit of PwMS around MS specialized nurse care and impact upon QoL (55% response rate from 1350 questionnaires), revealed that 83% of respondents preferred the care of their MS related issues from the MS nurse over other health professionals [84]. When people with early stage MS were surveyed as to whom they received the best education about their illness and coping strategies, despite an overall identified deficiency of education, the closest to ideal amount of education was received from MS specialist nurses [16]. For those with progressive MS, the MS specialized nurse was identified as a key person and coordinator of care in meeting an identified need to provide a more comprehensive and multidisciplinary approach to care, focusing on symptoms and QoL [6, 13, 61]. In 2015, a MS study found the establishment of proactive MS specialized nurse management in a primary-case based model with individualized care resulted in less emergency room visits and inpatient admissions over a 10 year period [85]. One prospective, randomized, controlled study assessed combined appointments with a multi-disciplinary MS Clinic team care, including a MS specialist nurse, vs standard care of referral to various specialists and allied health professionals as needed over a 6 month period for fifty moderately-disabled PwMS. They did not find differences in QoL measures, acknowledging that the overall care likely did not differ between arms, just in timing of visits [86].

This study provides an alternate and possibly cost-saving management strategy to complex management of symptoms and QoL measures for PwMS. These findings corroborate other studies identifying the benefits of NP patient care [27, 74, 75, 85], however, despite the magnitude of change in depression levels being statistically significant, it may not be to clinically meaningful levels. It is possible that there would have been greater change seen if participants had been primarily cared for by family physicians; 12.3% of 324 respondents in a recent survey of Canadians living with MS indicated that they received the majority of their care from general practitioners, 4% received no care for their MS, while 8% received their MS care primarily from walk-in clinics, after-hours clinics and emergencies [25].

### Limitations

The patients involved in this study self-reported their data which could be susceptible to subjective bias. Participants presumably participated in the study if they were open to being treated by an NP in addition to their CN, likely viewing NP positively, which could impact the finding of similar consultant satisfaction between arms. However, despite the potential bias, the findings of our study are consistent with what has been reported in the literature. Even though we opened the study to PwMS followed by general community neurologists or family physicians, all of the study participants were followed by a general neurologist. This is likely due to the methods of recruitment, primarily based within community neurologists’ office. Future studies could examine the value of an MS specialized NP in specialist and primary care settings. The control arm for our study included limited support to community neurologists by registered nurses who also work within a tertiary MS clinic setting. Due to the limited resources and time that the registered nurses could provide to the community neurologists, the registered nursing support consisted only of rooming patients, updating medication lists, and aiding in disease-modifying therapy access and renewals. Therefore, the registered nurses did not provide interventions provided by the nurse practitioner in the active arm of symptomatic strategies, lifestyle strategies etc. It is likely that a trained registered nurse could perform some of the duties performed by the NP in our study. However, as outlined in the study protocol paper in *Trials,* the NP provided an independent and holistic approach to study participants, including independent referrals to community resources, independent ordering of investigations and prescription of symptomatic medications [36]. Time was a limitation of this study, as 6 months may not be adequate time to see a significant change in a patient’s HADS, MFIS, or EQ5D. However, despite the short duration, this study showed an encouraging trend that NP-led care can positively influence or delay the decline of QoL measures such as depression for PwMS.

### Generalizability of study findings

Our study is generalizable to other healthcare settings, where generalists such as family doctors and general neurologists care for PwMS outside of a tertiary MS-subspecialty clinic. We did not examine the add-on aspects to NP-led care in the MS subspecialty clinic settings. We carried out our study with PwMS in a public healthcare system in Canada. However, there are similar challenges in meeting care needs of other chronic diseases in any healthcare system.

### Sex and gender

Our study did not select for participants, considering their sex and gender. Consecutive prospective participants visiting their community neurologists’ offices were approached to participate in the study. Prospective participants consented and were randomized after consent to participate in either arm. However, 82% of the participants were of female sex; this reflects the female preponderance to getting MS in comparison to males, and is consistent with other studies examining MS within populations. Gender was not collected in our study, and is a limitation of our study. Going forward, future clinical trials examining NP involvement in care for PwMS should examine if there are differences in consultant satisfaction by gender in receiving care from an NP.

## Conclusion

Our study demonstrated that adding a specialized NP to the care of PwMS improved depression over three and 6 months. There were no differences seen in satisfaction of care received by an NP as participants’ usual care. Examining the effect of a NP in a CN setting, delivering care to PwMS through multi-disciplinary means, such as through a patient advocacy management model [87], holds potential to improve symptoms for PwMS, improving healthcare access and satisfaction, and QoL for PwMS and their caregivers. In pressured public healthcare systems, alternate ways of supporting and treating PwMS need to be explored and supported to optimize the experience and functioning of PwMS.

## Supplementary Information

**Additional file 1.**

## Data Availability

All data used and analyzed during the current study are available from the corresponding author on reasonable request. Due to regulations from our Health Research Ethics Board at the University of Alberta around confidentiality and storage of the data obtained from the study, we are unable to share raw data in a public forum. Requests for raw data access will be reviewed for approval by the University of Alberta Research Ethics Board before release (Ethics approval number Pro00069595).

## Abbreviations

HADS-A: Hospital Anxiety and Depression Scale, Anxiety score
HADS-D: Hospital Anxiety and Depression Scale, Depression score
MS: Multiple Sclerosis
NP: Nurse practitioner
QoL: Quality of life
PwMS: People with MS
CSQ: Consultation Satisfaction Questionnaire
CN: Community neurologists
