# Supplementary material for: The effect of nurse practitioner (NP-led) care on health-related quality of life in people with multiple sclerosis – a randomized trial
Source: BMC Neurol. 2022 Jul 25;22:275. doi: 10.1186/s12883-022-02809-9 (PMC9310450; doi:10.1186/s12883-022-02809-9)
Supplement: Supplementary file 1 — Additional file 1. [file 12883_2022_2809_MOESM1_ESM.docx]

| **Appendix A Patients’ Satisfaction with care received (NP led arm vs Usual care arm)** | | | | | |
| --- | --- | --- | --- | --- | --- |
| Variable |  | Nurse Practitioner (%)  N = 118 | Community neurologist (%)  N = 108 | P-value | Total  N = 226 |
|  |  |  |  |  |  |
| Satisfaction Score | Mean (SD)  Median (IQR)  Range | 63.83 (5.63)  64.0 (60.0 – 68.0)  38.0 – 76.0  (n = 111) | 62.82 (5.45)  63.0 (60.0 – 67.0)  43.0 – 76.0  (n = 97) | 0.1935 | 63.36 (5.56)  63.0 (60.0 – 67.0)  38.0 – 76.0  (n = 208) |
|  |  |  |  |  |  |
| Nurse Practitioner was careful to check everything | 1.Strong Disagree | 1 (0.9) | 0 (0.0) | 0.5694 | 1 (0.48) |
|  | 2.Disagree | 0 (0.0) | 1 (1.02) |  | 1 (0.48) |
|  | 3.Netural | 11 (9.91) | 14 (14.29) |  | 25 (11.96) |
|  | 4.Agree | 41 (36.94) | 37 (37.76) |  | 78 (37.32) |
|  | 5.Strongly Agree | 58 (52.25) | 46 (46.94) |  | 104 (49.76) |
|  |  |  |  |  |  |
| Nurse listened carefully to what I had to say | 1.Strong Disagree | 1 (0.90) | 0 (0.0) | 0.4291 | 1 (0..48) |
|  | 2.Disagree | 0 (0.0) | 0 (0.0) |  | 0 (0.0) |
|  | 3.Netural | 7 (6.31) | 10 (10.20) |  | 17 (8.13) |
|  | 4.Agree | 38 (34.23) | 27 (27.55) |  | 65 (31.10) |
|  | 5.Strongly Agree | 65 (58.65) | 61 (62.24) |  | 126 (60.29) |
|  |  |  |  |  |  |
| I am totally satisfied with my visit to Nurse Practitioner | 1.Strong Disagree | 1 (0.90) | 1 (1.03) | 0.9219 | 2 (0.96) |
|  | 2.Disagree | 1 (0.90) | 1 (1.03) |  | 2 (0.96) |
|  | 3.Netural | 14 (12.61) | 10 (10.31) |  | 24 (11.54) |
|  | 4.Agree | 32 (28.32) | 24 (24.74) |  | 56 (26.92) |
|  | 5.Strongly Agree | 63 (56.76) | 61 (62.89) |  | 124 (59.62) |
|  |  |  |  |  |  |
| Nurse told me everything about my treatment | 1.Strong Disagree | 1 (0.90) | 0 (0.0) | 0.9111 | 1 (0.48) |
|  | 2.Disagree | 1 (0.90) | 1 (1.03) |  | 2 (0.96) |
|  | 3.Netural | 12 (10.81) | 13 (13.40) |  | 25 (12.02) |
|  | 4.Agree | 42 (37.84) | 33 (34.02) |  | 75 (36.06) |
|  | 5.Strongly Agree | 55 (49.55) | 50 (51.55) |  | 105 (50.48) |
|  |  |  |  |  |  |
| Nurse took notice of me as a person | 1.Strong Disagree | 1 (0.90) | 0 (0.0) |  | 1 (0.48) |
|  | 2.Disagree | 0 (0.0) | 2 (2.06) | 0.3041 | 2 (0.96) |
|  | 3.Netural | 11 (9.91) | 5 (5.15) |  | 16 (7.69) |
|  | 4.Agree | 28 (25.23) | 28 (28.57) |  | 56 (26.92) |
|  | 5.Strongly Agree | 71 (63.96) | 62 (63.92) |  | 133 (63.94) |
|  |  |  |  |  |  |
| I will follow Nurse advice because she/he is right | 1.Strong Disagree | 1 (0.90) | 0 (0.0) | 0.4957 | 1 (0.48) |
|  | 2.Disagree | 1 (0.90) | 4 (4.12) |  | 5 (2.40) |
|  | 3.Netural | 18 (16.22) | 13 (13.40) |  | 31 (14.90) |
|  | 4.Agree | 53 (47.75) | 44 (45.36) |  | 97 (46.63) |
|  | 5.Strongly Agree | 38 (34.23) | 36 (37.11) |  | 74 (35.58) |
|  |  |  |  |  |  |
| I am not completely satisfied with my visit to this Nurse | 1.Strong Disagree | 46 (41.44) | 44 (45.36) | 0.9601 | 90 (43.27) |
|  | 2.Disagree | 32 (28.83) | 28 (28.87) |  | 60 (28.65) |
|  | 3.Netural | 13 (11.71) | 11 (11.34) |  | 24 (11.54) |
|  | 4.Agree | 9 (8.11) | 7 (7.22) |  | 16 (7.69) |
|  | 5.Strongly Agree | 11 (9.91) | 7 (7.22) |  | 18 (8.65) |
|  |  |  |  |  |  |
| This Nurse was interested in me as a person not my illness in health of my whole family | 1.Strong Disagree | 3 (2.70) | 0 (0.0) | 0.5463 | 3 (1.44) |
|  | 2.Disagree | 1 (0.90) | 0 (0.0) |  | 1 (0.48) |
|  | 3.Netural | 17 (15.32) | 14 (14.43) |  | 31 (14.90) |
|  | 4.Agree | 35 (31.53) | 31 (31.96) |  | 66 (31.73) |
|  | 5.Strongly Agree | 55 (49.55) | 52 (53.61) |  | 107 (51.44) |
|  |  |  |  |  |  |
| I understand my illness about my family’s health much better after seeing this Nurse | 1.Strong Disagree | 1 (0.90) | 1 (1.02) | 0.7974 | 2 (0.96) |
|  | 2.Disagree | 2 (1.80) | 4 (4.08) |  | 6 (2.87) |
|  | 3.Netural | 37 (33.33) | 34 (34.96) |  | 71 (33.97) |
|  | 4.Agree | 37 (33.33) | 35 (35.71) |  | 72 (34.45) |
|  | 5.Strongly Agree | 34 (30.63) | 24 (24.49) |  | 58 (27.75) |
|  |  |  |  |  |  |
| This Nurse knows all about me | 1.Strong Disagree | 1 (0.90) | 3 (3.09) | 0.6360 | 4 (1.92) |
|  | 2.Disagree | 5 (4.50) | 6 (6.19) |  | 11 (5.29) |
|  | 3.Netural | 38 (34.23) | 29 (29.90) |  | 67 (32.21) |
|  | 4.Agree | 48 (43.24) | 38 (39.18) |  | 86 (41.65) |
|  | 5.Strongly Agree | 19 (17.12) | 21 (21.65) |  | 40 (19.23) |
|  |  |  |  |  |  |
| There are some things this Nurse does not know about me | 1.Strong Disagree | 8 (7.21) | 11 (11.34) | 0.5855 | 19 (9.13) |
|  | 2.Disagree | 23 (20.72) | 17 (17.53) |  | 40 (19.23) |
|  | 3.Netural | 31 (27.93) | 31 (31.96) |  | 62 (29.81) |
|  | 4.Agree | 45 (40.54) | 37 (38.14) |  | 82 (39.42) |
|  | 5.Strongly Agree | 4 (3.60) | 1 (1.03) |  | 5 (2.40) |
|  |  |  |  |  |  |
| I would find it difficult to tell this Nurse about some private things | 1.Strong Disagree | 26 (23.42) | 33 (34.02) | 0.1810 | 59 (28.37) |
|  | 2.Disagree | 50 (45.05) | 33 (34.02) |  | 83 (39.90) |
|  | 3.Netural | 19 (17.12) | 18 (18.56) |  |  |
|  | 4.Agree | 13 (11.71) | 13 (13.40) |  |  |
|  | 5.Strongly Agree | 3 (2.70) | 0 (0.0) |  |  |
|  |  |  |  |  |  |
| I felt able to tell this Nurse about personal things | 1.Strong Disagree | 2 (1.80) | 1 (1.03) | 0.3854 | 3 (1.44) |
|  | 2.Disagree | 3 (2.70) | 3 (3.09) |  | 6 (2.88) |
|  | 3.Netural | 15 (13.51) | 22 (22.68) |  | 37 (17.79) |
|  | 4.Agree | 59 (53.15) | 41 (42.27) |  | 100 (48.08) |
|  | 5.Strongly Agree | 32 (28.83) | 30 (30.93) |  | 62 (29.81) |
|  |  |  |  |  |  |
| I felt this Nurse really knew what I was thinking | 1.Strong Disagree | 1 (0.90) | 1 (1.03) | 0.4058 | 2 (0.96) |
|  | 2.Disagree | 7 (6.31) | 6 (6.19) |  | 13 (6.25) |
|  | 3.Netural | 37 (33.33) | 40 (41.24) |  | 77 (37.02) |
|  | 4.Agree | 54 (48.65) | 35 (36.08) |  | 89 (42.79) |
|  | 5.Strongly Agree | 12 (10.81) | 15 (15.46) |  | 27 (12.98) |
|  |  |  |  |  |  |
| I wish it had been possible to spend a little longer with this Nurse | 1.Strong Disagree | 3 (2.70) | 4 (4.12) | 0.7733 | 7 (3.37) |
|  | 2.Disagree | 25 (22.52) | 23 (23.71) |  | 48 (23.08) |
|  | 3.Netural | 42 (37.84) | 41 (42.27) |  | 83 (39.90) |
|  | 4.Agree | 31 (27.93) | 24 (24.74) |  | 55 (26.44) |
|  | 5.Strongly Agree | 10 (9.01) | 5 (5.15) |  | 15 (7.21) |
|  |  |  |  |  |  |
| The time I was able to spend with this Nurse was a bit short | 1.Strong Disagree | 11 (9.91) | 13 (13.40) | 0.3988 | 24 (11.54) |
|  | 2.Disagree | 53 (47.75) | 48 (49.48) |  | 101 (48.56) |
|  | 3.Netural | 24 (21.62) | 24 (24.74) |  | 48 (23.08) |
|  | 4.Agree | 20 (18.02) | 12 (12.37) |  | 32 (15.38) |
|  | 5.Strongly Agree | 3 (2.70) | 0 (0.0) |  | 3 (1.44) |
|  |  |  |  |  |  |
| The time I was able to spend with this Nurse was not long enough to deal with everything I wanted | 1.Strong Disagree | 13 (11.71) | 19 (19.59) | 0.2551 | 32 (15.38) |
|  | 2.Disagree | 54 (48.65) | 45 (46.39) |  | 99 (47.60) |
|  | 3.Netural | 25 (22.52) | 24 (24.74) |  | 49 (23.56) |
|  | 4.Agree | 17 (15.32) | 9 (9.28) |  | 26 (12.50) |
|  | 5.Strongly Agree | 2 (1.80) | 0 (0.0) |  | 2 (0.96) |
|  |  |  |  |  |  |
| Some things about the consultation with the Nurse could have been better | 1.Strong Disagree | 21 (18.92) | 26 (26.80) | 0.3749 | 47 (22.60) |
|  | 2.Disagree | 54 (48.65) | 36 (37.11) |  | 90 (43.27) |
|  | 3.Netural | 23 (20.72) | 26 (26.08) |  | 49 (23.56) |
|  | 4.Agree | 11 (9.91) | 8 (8.25) |  | 19 (9.13) |
|  | 5.Strongly Agree | 2 (1.80) | 1 (1.03) |  | 3 (1.44) |

Note: Chi-Square test and Fisher test (*) were used for categorical variables; T-test was used for continuous variables;
